# Identification and detection of *iha* subtypes in LEE-negative Shiga toxin-producing *Escherichia coli* (STEC) strains isolated from humans, cattle and food

**DOI:** 10.1016/j.heliyon.2019.e03015

**Published:** 2019-12-11

**Authors:** Rocío Colello, Alejandra Krüger, María Victoria Velez, Felipe Del Canto, Analía Inés Etcheverría, Roberto Vidal, Nora Lía Padola

**Affiliations:** a Laboratorio de Inmunoquímica y Biotecnología, Centro de Investigación Veterinaria de Tandil (CIVETAN), CONICET, CICPBA, Facultad de Ciencias Veterinarias, UNCPBA, Tandil, Argentina; b Programa de Microbiología y Micología, Instituto de Ciencias Biomédicas, Facultad de Medicina, Universidad de Chile, Santiago, Chile; c Instituto Milenio de Inmunología e Inmunoterapia, Facultad de Medicina, Universidad de Chile, Santiago, Chile

**Keywords:** Microbiology, Food technology, Food microbiology, Animal behavior, Public health, Infectious disease, STEC iha subtype genomics PCR design

## Abstract

LEE-negative Shiga toxin-producing *Escherichia coli* (STEC) strains are important cause of infection in humans and they should be included in the public health surveillance systems. Some isolates have been associated with haemolytic uremic syndrome (HUS) but the mechanisms of pathogenicity are is a field continuos broadening of knowledge. The IrgA homologue adhesin (Iha), encoded by *iha*, is an adherence-conferring protein and also a siderophore receptor distributed among LEE-negative STEC strains. This study reports the presence of different subtypes of *iha* in LEE-negative STEC strains. We used genomic analyses to design PCR assays for detecting each of the different *iha* subtypes and also, all the subtypes simultaneously. LEE-negative STEC strains were designed and different localizations of this gene in STEC subgroups were examinated.

Genomic analysis detected *iha* in a high percentage of LEE-negative STEC strains. These strains generally carried *iha* sequences similar to those harbored by the Locus of Adhesion and Autoaggregation (LAA) or by the plasmid pO113. Besides, almost half of the strains carried both subtypes. Similar results were observed by PCR, detecting *iha* LAA in 87% of the strains (117/135) and *iha* pO113 in 32% of strains (43/135). Thus, we designed PCR assays that allow rapid detection of *iha* subtypes harbored by LEE-negative strains. These results highlight the need to investigate the individual and orchestrated role of virulence genes that determine the STEC capacity of causing serious disease, which would allow for identification of target candidates to develop therapies against HUS.

## 1 Introduction

Shiga toxin-producing *Escherichia coli* (STEC) is an important group of pathogens which cause serious human disease, including bloody diarrhea and haemolytic uraemic syndrome (HUS) [18, 23].

STEC are classified into two major groups in accordance with the presence of the locus of enterocyte effacement (LEE). LEE-positive strains have the ability to produce attaching and effacing lesions on the intestinal epithelium [23]. However, the presence of LEE is not essential for pathogenesis of all the STEC strains since some LEE-negative STEC strains have been also associated with severe disease in humans [4]. The majority of STEC strains associated with disease in humans adhere to the intestinal epithelial cells [12] because the adhesion presumably allows the pathogens to deliver toxins efficiently to the host [33]. This colonization ability is often linked to the expression of specific mechanisms. However, little is known about the adherence mechanisms of LEE-negative STEC strains to epithelial cells [36].

Among STEC, a range of novel adhesins have been identified, including Iha. It was first described as an adhesin in a STEC O157:H7 strain, and it was named “IrgA homologue adhesin” due to homology shared with the IrgA of *Vibrio cholerae* [33]. The *iha* gene was found in duplicated genomic islands (called OI-43 and OI-48), which encode Tellurite resistance (Te^r^), AidaA-1, and Iha [37].

Interestingly, *iha* has been detected in LEE-positive STEC and LEE-negative STEC, as well as in uropathogenic *E. coli* [14, 30, 33]. Moreover, LEE-negative STEC strains, *iha* is harbored by mobile genetic elements that encode other virulence factors involved in human pathogenicity, namely plasmids and pathogenicity islands (PAIs). For example, plasmid pO113 encodes several toxins and adhesins such as EhxA, Saa, SubAB, and Iha [15, 24]. The PAI Locus of Proteolysis Activity (LPA) encodes EspI and Iha [30]. A novel PAI, named Locus of Adhesion and Autoaggregation (LAA) is found either as a “complete” structure with four modules: module I (*hes* and other genes), module II (*iha*, *lesP* and others genes), module III (*pagC*, *tpsA*, and other genes), and module IV (*agn43* and other genes); or as an “incomplete” structure if one of the modules is missing (<4 modules) [17].

Although some studies have found and evaluated the presence of the different *iha* subtypes, this information in LEE-negative STEC strains is scarce. Therefore, our objetives are to design PCR assays for detecting each of the different subtypes of *iha* in LEE-negative STEC strains and to examine the different localizations of this gene in STEC subgroups. Even more, and stemming from these objetives, we aimed at designing a general PCR assay for detecting all subtypes of *iha* in LEE-negative STEC strains.

## 2 Materials and methods

### 2.1 Genomic analysis

Thirty LEE-negative STEC strains were selected for the sequence analysis. Fourteen strains were isolated from dairy, beef cattle and food in Argentina [21, 29] and 16 strains from cattle and humans in Chile [16]. Draft genomes of all of these strains were previously obtained [16]. The accession number of the corresponding sequences are shown in Table 1.

**Table 1:** Strains ID and accession numbers (NCBI nucleotide) for draft genomic sequences included in this study.

Strain ID	Serogroup/Serotype	Origin	Country	Year of isolation	Accession Number	*In silico iha* subtype
CM 15-2	O8:H16	Ground beef	Argentina	1998	QESP00000000	*iha* pO113
30M	O8:H19	Ground beef	Argentina	1998	QESO00000000	-
45-2-4	O8:H19	Cattle	Argentina	2009	QESN00000000	-
HT 1-6	O20:H19	Hamburguer	Argentina	1998	QESL00000000	*iha* LAA/*iha* pO113
HW 1-3	O22:H8	Hamburguer	Argentina	1998	QESK00000000	*iha* LAA/ *iha* pO113
AM 162-1	O39:H49	Cattle	Argentina	1998	QESJ00000000	*iha* LAA/ *iha* pO113
V07-4-4	O91:H21	Cattle	Argentina	2008	QESH00000000	*iha* LAA/ *iha* pO113
AP 16-1	O91:H21	Cattle	Argentina	1998	QESG00000000	*iha* LAA/ *iha* pO113
47-1-1	O91:H21	Cattle	Argentina	2009	QESF00000000	*iha* LAA/ *iha* pO113
FO 130	O91:H21	Cattle	Argentina	2001	QESE00000000	*iha* LAA/*iha* pO113
5-1-1	O91:H21	Cattle	Argentina	2010	QESD00000000	*iha* LAA/ *iha* pO113
AP 32-1	O117:H7	Cattle	Argentina	1998	QERR00000000	*iha* LAA
AP 31-1	O141:H8	Cattle	Argentina	1998	QERO00000000	*iha* LAA/ *iha* pO113
180-3-4r	O178:H19	Chicken burguer	Argentina	2007	QERJ00000000	*iha* LAA
26_1	O2	Cattle	Chile	NA	QESR00000000	*iha* EDL933
365_1	O7	Cattle	Chile	NA	QESQ00000000	*iha* LAA/ *iha* pO113
116_1	O20:H19	Cattle	Chile	NA	QESM00000000	*iha* LAA/ *iha* pO113
348_3	O46	Cattle	Chile	NA	QESI00000000	*iha* LAA/ *iha* pO113
211_1	O103:H42	Cattle	Chile	NA	QESC00000000	*iha* LAA/ *iha* pO113
E044-00	O113:H21	Human	Chile	2000	QERZ00000000	*iha* LAA
E045-00	O113:H21	Human	Chile	2000	QERY00000000	*iha* LAA
E042-00	O113:H21	Human	Chile	2000	QERU00000000	*iha* LAA
E043-00	O113:H21	Human	Chile	2000	QERT00000000	*iha* LAA
E046-00	O113:H21	Human	Chile	2000	QERS00000000	*iha* LAA
6_6	O130:H11	Cattle	Chile	NA	QERQ00000000	*iha* pO113
208_3	O139:H19	Cattle	Chile	NA	QERP00000000	*iha* pO113
175_1	O156:H-	Cattle	Chile	NA	QERN00000000	*iha* LAA
218_8	O163:H9	Cattle	Chile	NA	QERM00000000	*iha* LAA/ *iha* pO113
115_4	O171:H2	Cattle	Chile	NA	QERL00000000	*iha* LAA
58_3	O174:H21	Cattle	Chile	NA	QERK00000000	*iha* LAA

### 2.2 *In silico* identification of *iha* subtypes

The presence/absence of *iha* genes and their localization were determined by using VirulenceFinder and BLAST programs [2, 10]. Open reading frames (ORFs) were detected by using the ORFfinder program [28]. The identified nucleotide sequences of *iha* were downloaded from the GenBank. A multiple alignment and phylogenetic relathionship of nucleotide sequences of *iha* were performed by using MUSCLE in the Ugene software, thus generating a maximum-likelihood phylogenetic tree [20].

Genomes of STEC strains were annotated by using RAST (Rapid Annotation using Subsystem Technology) server [3] and, when possible, the regions near the *iha* gene were manually analyzed.

### 2.3 Polymerase chain reaction PCR detection of *iha* genes

Primers were designed to amplify *iha* (*iha* without discrimination of any subtype) and also specific *iha* subtypes (named *iha* pO113 and *iha* LAA). DNA was extracted by following methodologies previously described by Parma et al. [22]. Amplification was performed in a total volume of 25 μl. The reaction mixture contained 500 mM KCl, 100 mM Tris–HCl pH 9, Triton X-100, 25 mM MgCl_2_, 200 mM of each deoxynucleotide (dATP, dGTP, dCTP, dTTP), 1U TaqDNA Polymerase and 2.5 ul DNA. Primers and PCR conditions are described in Table 2.

**Table 2:** Primers used for *iha* detection and size of PCR amplicons.

Primer	Sequence (5′-3′)	Size (bp)	Reference
pO113 *iha* F	GGCACTGAGATCAGTGGAGG	600	This study
pO113 *iha* R	ACCAGAGCATATCTTGTTCCG		
*iha* general F	AACTGGCAGATCACCGAAGA	346	This study
*iha* general R	GCGACATCCAGTAATTTCGCT		
LAA *iha* F	TTTCAGCCAGCAGCATGGCA	172	[7]
LAA *iha* R	ACATCCACACCCTCCACAGC		

A total of 135 LEE-negative STEC strains were screened. These STEC strains were isolated from dairy and beef cattle and food (beef, ground beef, hamburguer and chicken burger) between 2000 and 2015 from Argentina. In previous studies, these isolates were analyzed for the presence of *vt1*, *vt2*, *eae, ehx*A, and *saa* genes by PCR, and O and H types were determined by the microagglutination technique (Table 3) [1,8,21,22]. Twenty eight sequenced LEE-negative STEC strains, whose genomes were previously sequenced [16], harboring different *iha* subtypes were used as positive controls. In addition, other two sequenced LEE-negative and *iha* negative STEC strains (30M and CM15-2) and 69 LEE-positive STEC strains belonging to serotypes O26:H11, O157:H7, O111:H-, O145:H-, and O103:H-, were used as negative controls.

**Table 3:** Distribution of *iha* genes, virulence profile and serogroup of LEE-negative STEC strains isolated from different origins.

Serotype	*iha* pO113	*iha* LAA	*iha* general	Virulence Profile	Origin
O8:H16 (1)	+	-	+	*vt1, saa*	Ground beef
O8:H19 (2)	-	-	-	*vt2*	Cattle
O20:H19 (1)	+	+	+	*vt1, vt2, ehxA, saa*	Hamburguer
O22:H8 (1)	-	+	+	*vt1, vt2, ehxA, saa*	Hamburguer
O39:H49 (1)	+	+	+	*vt2, ehxA, saa*	Cattle
O91:H21 (10)	+	+	+	*vt2, saa, ehxA*	Cattle
O91:H21 (4)	-	+	+	*vt2, saa, ehxA*	Cattle
O91:H21 (4)	+	+	+	*vt2, ehxA*	Cattle
O91:H21 (1)	-	+	+	*vt2, ehxA*	Cattle
O91:H21 (1)	-	+	+	*vt1, ehxA*	Cattle
O91:H40 (1)	-	+	+	*vt2, ehxA*	Chicken burger
O91:H28 (1)	-	+	+	*vt2, saa, ehxA*	Cattle
O91:H21 (1)	+	+	+	*vt2*	Cattle
O103:H26 (1)	-	+	+	*vt2*	Beef
O103:H42 (1)	-	+	+	*vt2*	Beef
O113:H21 (10)	+	+	+	*vt2, saa, ehxA*	Cattle
O113:H21 (4)	-	+	+	*vt2, saa, ehxA*	Cattle
O113:H21 (2)	-	+	+	*vt2, saa, ehxA*	Chicken burger
O113:H21 (1)	+	+	+	*vt1, vt2, ehxA, saa*	Cattle
O117:H7 (1)	-	+	+	*vt2, saa*	Cattle
O130:H11 (23)	-	+	+	*vt1, vt2, ehxA, saa*	Cattle
O130:H11 (6)	+	+	+	*vt1, vt2, ehxA, saa*	Cattle
O130:H11 (2)	+	+	+	*vt1, vt2, ehxA, saa*	Chicken burger
O130:H11 (1)	+	+	+	*vt1, ehxA, saa*	Cattle
O141:H8 (1)	-	+	+	*vt1, vt2, ehxA, saa*	Cattle
O174:H21 (9)	-	-	-	*vt2*	Cattle
O174:H21 (12)	-	+	+	*vt2*	Cattle
O174:H21 (1)	-	+	+	*vt1, vt2, ehxA, saa*	Cattle
O174:H21 (1)	-	+	+	*vt2, saa*	Cattle
O178:H19 (6)	-	-	-	*vt2*	Cattle
O178:H19 (1)	+	+	+	*vt2, ehxA, saa*	Cattle
O178:H19 (4)	+	+	+	*vt1, vt2, ehxA,saa*	Cattle
O178:H19 (13)	-	+	+	*vt2*	Cattle
O178:H19 (1)	-	+	+	*vt2, ehxA,saa*	Cattle
O178:H19 (1)	-	+	+	*vt1, vt2, ehxA,saa*	Cattle
O178: H21 (2)	-	+	+	*vt2*	Cattle
O178: H25 (1)	-	+	+	*vt2, ehxA, saa*	Cattle
O178:H28 (1)	-	+	+	*vt2, ehxA, saa*	Cattle

### 2.4 Statistical analysis

The association between LEE-negative STEC strains and *iha* gene subtype was analyzed by using the Fisher's test with a confidence level of 95%.

## 3 Results

### 3.1 Genomic analysis

Genomes of thirty LEE-negative STEC strains isolated in Argentina (n = 14) and Chile (n = 16) were analyzed. The presence of one or two *iha* genes was detected in 28/30 (93%) of the STEC genomes by using VirulenceFinder tool and BLAST. The *iha* nucleotide sequences identified were similar to *iha* genes carried by STEC strain B2F1 (LAA pathogenicity island), 4797/97 (LPA pathogenicity island), 98NK2 (plasmid pO113) and EDL933 (accession numbers AFDQ01000026.1, AJ278144.1 and AF399919, AE005174, respectively). The comparative analysis showed that *iha* of LAA and *iha* of LPA are 99.7% similar. Therefore, in this study, we named the detected *iha* subtypes as *iha* LAA, *iha* pO113 and *iha* EDL933.

### 3.2 *In silico* identification of *iha* subtypes

Among the 28 STEC genomes positive for *iha*, 14 (47%) carried both *iha* LAA and *iha* pO113 subtypes*,* 12 (40%) *iha* LAA, 3 (10%) *iha* pO113, and 1 (3%) *iha* EDL933. In particular, 14 (70%) and 16 (80%) out of 20 STEC strains isolated from cattle were positive to *iha* LAA and *iha* pO113, respectively; and only one was positive for *iha* EDL933. Three (60%) out of 5 and 3 (60%) of STEC strains isolated from food were positive to *iha* LAA and *iha* pO113, respectively. All of the LEE-negative STEC obtained from humans were positive for the presence of *iha* LAA.

**Figure 1:**
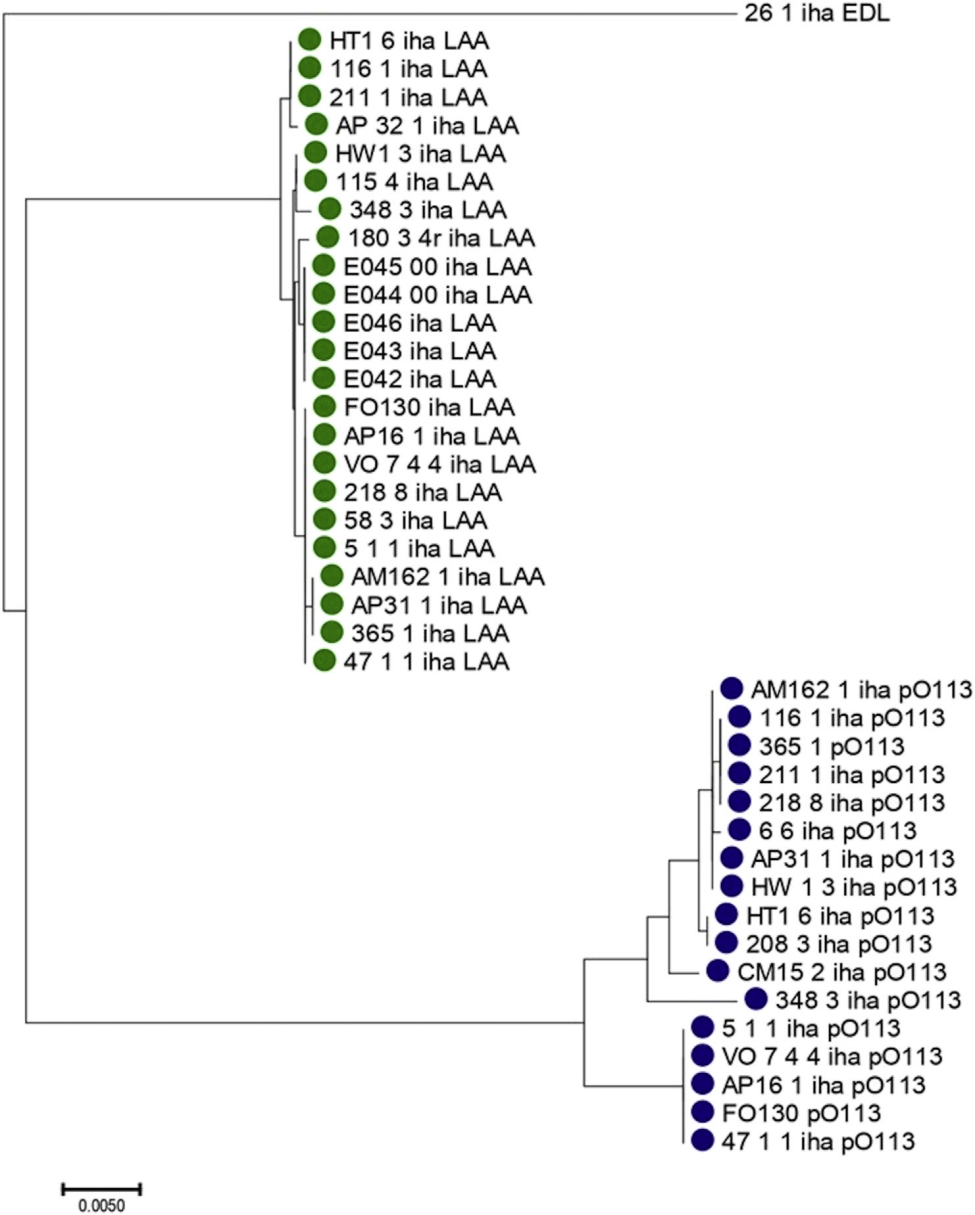
Phylogenetic tree based on *iha* sequences.

A phylogenetic tree based on *iha* sequences showed three clades, each one corresponding to the subtypes *iha* LAA, *iha* pO113, and *iha* EDL933, respectively (Figure 1). In clade *iha* pO113, sequences shared near 98% similarity. In clade *iha* LAA, the sequences shared near 99% sequence similarity and 94% similarity with clade *iha* pO113. We found that one *iha* EDL933 shared 91% similarity with clades *iha* pO113 and *iha* LAA.

Once the contigs carrying *iha* genes were selected, annotated regions near *iha* were examined when they were available. Thus, we identified three different genetic contexts for each *iha* subtype: first, genes encoding AtoS, AtoC, acetoacetyl-CoA transferases and a ShiA homologue were generally identified upstream *iha* gene, while genes encoding the vitamin B12 receptor BtuB, a N-acetylgalactosamine-sulfatase and a putative porin gene were detected downstream, in a LAA island context. Second, an entry exclusion protein coding gene was identified in the proximity of *iha* pO113. Additionally, replication initiation proteins (Rep) and other plasmid element-coding genes, as well as *saa* and/or *subA* genes, could be identified in several contigs carrying *iha* pO113. Finally, only one small contig carried *iha* EDL933 that had a region encoding tellurite resistance proteins, was observed.

### 3.3 Distribution of *iha* by PCR detection

Among the 135 LEE-negative STEC strains screened by PCR, 124 (92%) were positive for *iha* (*iha* general) (Table 3). Specific detection of *iha* LAA showed that this subtype was predominant among LEE-negative STEC strains (117/135, 87%) and detected in several serotypes. The *iha* pO113 subtype was detected in 32% of the strains (43/135). The *iha* LAA and *iha* pO113 subtypes were significantly associated with LEE-negative STEC strains (both with p > 0.00001).

## 4 Discussion

In recent years, there has been an increasing interest in the study and characterization of LEE-negative STEC strains because some isolates have been associated with HUS [32]. Iha is an adherence-conferring protein and also a siderophore receptor that is distributed among STEC strains of a variety of serotypes [34] and was reported in PAIs and plamids of LEE-negative STEC [17, 25].

In this study, genomic analysis and PCR assays detected *iha* subtypes in a high percentage of LEE-negative STEC strains. In other studies, such as Toma et al. [34] the most prevalent adhesin gene found among all LEE-negative and LEE-positive STEC strains was *iha*; 127 out of 139 strains (91%) from humans (54), animals (52), and food (33). Similarly, Miko et al. [15] found that *iha* was common in O178 LEE-negative STEC strains serogroup tested. According to Cáceres et al. [5] *iha* showed a high distribution in strains isolated from calves and adults (87.04 and 98.48%, respectively). On the other hand, a study on distribution of gene markers for OI-43/48 detected *iha* in 45% of LEE-positive STEC strains serogroup O103 [13]. However, we should be cautious about the interpretation of these results because only one subtype of *iha* was positively screened. Taking into account different *iha* subtypes are associated to LEE-positive or LEE-negative STEC strains, subtype-specific detection should be performed.

Interestingly, the genomic analysis allowed for the identification of particular characteristics of *iha* genes in 30 LEE-negative STEC strains. These strains generally carried *iha* sequences similar to those encoded by LAA or pO113, here named *iha* LAA and *iha* pO113 subtypes. Besides, almost half of the strains carried both subtypes simultaneously. Similar results were observed by PCR analyses that detected 87% of strains (117/135) from *iha* LAA and 32% of strains (43/135) from *iha* pO113.

Aligment and phylogenetic analysis revealed that *iha* LAA and *iha* pO113 subtypes were highly similar, whereas they have lower sequence similarity regarding *iha* gene in STEC EDL933. These results suggest that *iha* genes from LEE-negative and LEE-positive STEC strains may have different origins and are in agreement with those previously reported by Ju et al. [11], who reported that *iha* genes from LEE-positive STEC had high similarity (99.6%), whereas they had lower sequence similarity (91.1–93.6%) than *iha* genes from LEE-negative STEC. The scientific sustenance for the apparent differences in virulence between different serotypes is not known. Furthermore, evolution of PAIs and plasmids can occur by several processes, including recombination events, leading to deletion or acquisition of DNA, and horizontal transferance events that determine separate evolution and sequence divergence [31].

The DNA sequences upstream and downstream of the *iha* LAA subtype were highly similar among the positive strains and to the same regions in LAA of STEC B2F1, suggesting that this subtype is generally located in this pathogenicity island. On the other hand, elements and genes identified in contigs carrying *iha* pO113 indicate that this subtype is located in plasmids. Bacteria express numerous surface structures that enable them to interact with and survive in changing environments. In addition, iron uptake systems are vital to bacterial survival within the host but mucosal surfaces of the host cells are iron-poor environments [27]. Many bacteria secrete siderophores which bind iron and are brought into the bacterial cell via a specific siderophore receptor. The capacity of Iha to transport siderophores is TonB-dependent. The TonB protein, which is anchored in the cytoplasmic membrane, provides energy to the outer membrane receptors for the transport of iron compounds [14, 26, 35]. Interestingly, our study found that *iha* LAA is located in the proximity to *btuB* gene which encodes an outer membrane protein required for vitamin B12 uptake and is also TonB-dependent [6].

The virulence of STEC is dependent on its ability to multiply in host tissues [35]. Iha is common in all seropathotypes, suggesting that it is a necessary but not sufficient adhesin for human infection [34]. However, mutations of TonB-dependent iron transport systems have been assessed with several pathogens resulting in an avirulent phenotype in animal models [35]. Moreover, it has been reported that during iron shortage, siderophore synthesis and expression of siderophore transporters increase, thus eliciting an enhanced immune response against these antigens and may be used as the basis for development of a vaccine candidate for STEC control [9].

In conclusion, the capacity of LEE-negative STEC strains to cause life-threatening human disease has been long-recognized [19]. Our study showed that LEE-negative STEC strains frequently had one or two *iha* genes located in mobile elements that differed in sequence with the *iha* gene present in different localizations described previously. Further studies are needed that shed light on the actual role of Iha in STEC pathogenesis and find out how the combination of different genes determine STEC virulence leading to serious disease, a repertoire that could be the basis for developing therapies against HUS. In the meantime, we designed PCR assays that may contribute to the rapid detection of *iha*, based on common sequences among subtypes, and specific *iha* subtypes for future basic and/or epidemiological studies.

## Declarations

### Author contribution statement

Colello R: Conceived and designed the experiments; Performed the experiments; Analyzed and interpreted the data; Wrote the paper.

Krüger A: Conceived and designed the experiments; Performed the experiments; Analyzed and interpreted the data.

Velez MV: Performed the experiments.

Del Canto F, Vidal R: Analyzed and interpreted the data.

Etcheverría AI: Performed the experiments; Contributed reagents, materials, analysis tools or data.

Padola NL: Analyzed and interpreted the data; Contributed reagents, materials, analysis tools or data.

### Funding statement

This work was supported by PICT 2015–2666, CICPBA from Argentina and FONDECYT grants 1161161 and 11150966 from Chile.

### Competing interest statement

The authors declare no conflict of interest.

### Additional information

No additional information is available for this paper.
